# Secukinumab for the treatment of generalized pustular psoriasis: A case report

**DOI:** 10.1097/MD.0000000000033693

**Published:** 2023-05-05

**Authors:** Jiayan Li, Xue Gao, Lili Ma, Yimiao Fang

**Affiliations:** a Zhejiang Chinese Medical University, Hangzhou, China; b The First Affiliated Hospital of Zhejiang Chinese Medical University, Hangzhou, China.

**Keywords:** case report, generalized pustular psoriasis, secukinumab, systemic treatment

## Abstract

Generalized pustular psoriasis (GPP) is a rare subtype of psoriasis that manifests as a generalized pustular eruption.

**Patient concerns:**

A 31-year-old female was admitted to the hospital in June 2021 with a widespread erythematous, itchy, and scaly rash for a week. The patient has a 10-year history of psoriasis vulgaris.

**Diagnosis:**

GPP complicated by a late-stage viral infection and early-stage renal damage.

**Interventions:**

Weekly subcutaneous injections of 300 mg of secukinumab for a month, followed by monthly (every 4 weeks) injections of 300 mg of secukinumab for 20 weeks.

**Outcomes:**

The symptoms of pustules and erythema were reduced, and the patient reported pain relief soon after the first injection. The patient had no serious adverse reactions during treatment and follow-up.

**Conclusions:**

Secukinumab might be an optional treatment strategy for GPP.

## 1. Introduction

Generalized pustular psoriasis (GPP) manifests as widespread inflamed, erythematous skin with an acute or subacute eruption of pustules.^[1]^ The clinical course of GPP is not predictable, and patients can go through periods of disease exacerbation and dormancy. GPP can lead to fatal complications, including sepsis, neutrophilic pneumonitis, neutrophilic cholangitis, and acute respiratory distress syndrome.

Continued therapy is usually necessary to avoid the reoccurrence of flares in GPP. However, it can be difficult to determine the best treatment strategy due to a lack of high-quality data on the management of GPP. Traditional therapies for GPP include systemic acitretin, methotrexate, and cyclosporine.^[2]^ However, these drugs are limited by their slow onset and severe side effects. Secukinumab is a recently developed anti-IL-17 biologic which has shown good efficacy in the treatment of many patients with pustular psoriasis. This case report describes a GPP patient treated with secukinumab.

## 2. Case presentation

A 31-year-old female patient came to our hospital in June 2021 with a widespread erythematous, itchy, and scaly rash for a week. Her symptoms were aggravated after the use of ointment of unknown origin and black soybean tar by herself. The patient had a 10-year history of psoriasis, which was not adequately addressed and treated. The patients once received hormones to control the rash and compound glycyrrhizin tablets to control inflammation, dehydration, and other symptoms 2 years ago. After the symptoms improved, the patient took traditional Chinese medicine orally for a long time, and her condition was stable before this disease attack. The patient has no family history of psoriasis.

**Figure 1.**
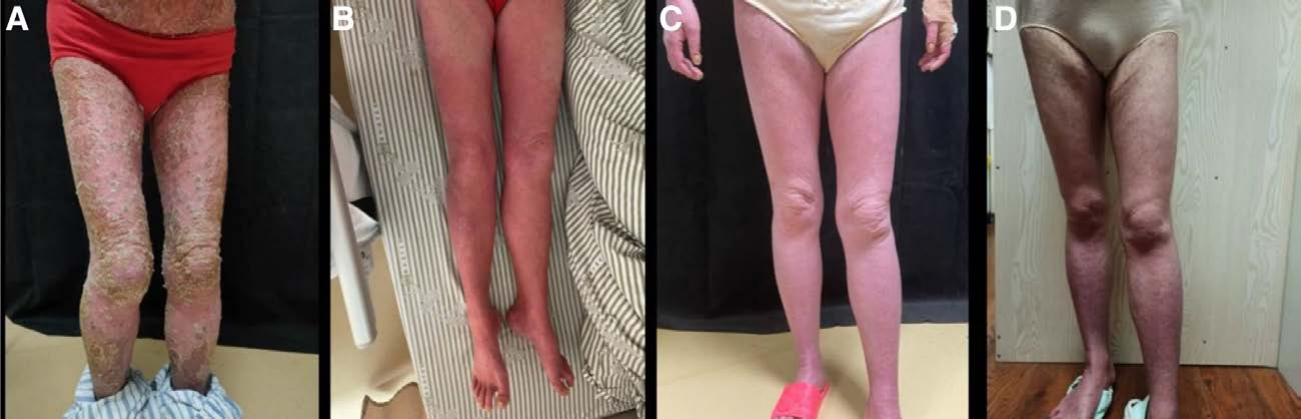
Condition of the lower extremities of the patient at (A) baseline upon admission, (B) 3 d after the first secukinumab injection, (C) 1 d before the third injection, and (D) 8 wk after the first injection.

On admission, the patient had diffuse erythema all over the body, and the feet, hands, and face were heavier. In addition, white scales were covered, and a thin film phenomenon was observed after the scales were scraped off, and needle-like pus was covered and partially fused into sheets (Fig. 1A). The skin lesions were painful and prevented the patient from sitting or reclining comfortably. A diagnosis of GPP was made according to the symptoms and medical history.

The patient’s laboratory results led to a diagnosis of viral infection and early renal damage, which was treated by the nephropathy department (Table S1, Supplemental Digital Content, http://links.lww.com/MD/I916). Skin wound ulcer and drug susceptibility testing showed a *Staphylococcus aureus* infection, which was treated with levofloxacin injection.

To treat GPP, the patient received a standard psoriasis treatment regimen of weekly subcutaneous injections of 300 mg of secukinumab (Secukinumab for injection [Costantine], Novartis) in weeks 0 to 4, followed by injections of 300 mg of secukinumab every 4 weeks for 20 weeks.

Three days after the first injection of secukinumab, the pustules and erythema were significantly reduced, and the patient reported pain relief (Fig. 1B). One week after the first injection of secukinumab, redness was still visible on the skin of both lower extremities, but the pustules continued to decrease. One day after the second injection, there was no new rash on any part of the body, and the facial rash basically subsided. One week after the second injection, the erythema of the whole body dimmed out, the scales were reduced, and the patient reported no discomfort, such as pain and itching (Fig. 1C). Eight weeks after the first injection, the whole body rash darkened without obvious scaling and infiltration (Fig. 1D). The patient was followed up for 6 months and had no recurrence of GPP or serious adverse reactions during treatment and follow-up.

## 3. Discussion

This study reported a 31-year-old female patient with a 10-year history of psoriasis vulgaris who never underwent systemic treatment and later developed GPP. Combined with the descriptions made by the patient, the physicians hypothesized that the patient’s irregular history of hormonal medication could be the trigger. At admission, there was a late-stage viral infection and early renal damage combined with the patient’s medical history and treatment history. Given the patient’s condition, the physician’s experience, and available guidelines, secukinumab was selected for treatment, despite the fact that infection is an adverse reaction of secukinumab. The patient was treated with secukinumab for 20 weeks and followed up for 6 months without recurrence or serious adverse reactions.

The findings, in this case, corroborate those of a phase III study in Japan, in which 12 patients with GPP were treated with secukinumab alone or in combination for 12 weeks; most of the patient’s symptoms were improved and maintained until 52 weeks.^[3]^ In another case series of 4 children, off-label treatment with secukinumab almost completely resolved the GPP lesions in all 4 patients after 1 month of treatment. In Japan, the latter evidence has led to the inclusion of secukinumab in the treatment guidelines for pustular psoriasis.^[4]^

In the case reported here, the pustules, erythema, and pain were significantly reduced as early as 3 days after the first injection of secukinumab. In the phase III trial by Imafuku et al,^[3]^ the earliest assessment after starting secukinumab was 7 days, and 4 (36.4%) patients were very much improved, and 5 (45.5%) patients were much improved, supporting the early onset observed in the present study. A response in the psoriasis area-and-severity index score was also observed as early as 1 week in a pooled analysis of 2 phase III trials.^[5]^

In conclusion, secukinumab might be an optional treatment for GPP. The symptoms of the reported patient with viral infections and early renal damage were improved after being treated with secukinumab without any serious adverse events.

## Acknowledgments

Thank the patient for the informed consent to this study.

## Author contributions

**Conceptualization:** Jiayan Li, Xue Gao, Yimiao Fang.

**Formal analysis:** Jiayan Li, Xue Gao, Yimiao Fang.

**Writing – original draft:** Jiayan Li, Lili Ma.

**Writing – review & editing:** Lili Ma.

## Supplementary Material


