# Supplementary material for: Secukinumab for the treatment of generalized pustular psoriasis: A case report
Source: Medicine (Baltimore). 2023 May 5;102(18):e33693. doi: 10.1097/MD.0000000000033693 (PMC10158903; doi:10.1097/MD.0000000000033693)
Supplement: Supplementary file 1 [file medi-102-e33693-s001.pdf]

Supplementary Table 1 Laboratory results of viral infection and renal damage

Characteristics	On admission	One week after admission	Two months after admission
White blood cell count, /L	11.9×10^{9}	9.3×10^{9}	/
Neutrophil count, /L	9.8×10^{9}	7.5×10^{9}	/
Erythrocyte sedimentation rate, mm/h	40	/	/
Cytomegalovirus IgG, AU/mL	> 250.00	Normal	/
Herpes simplex virus type I IgG	Positive	Negative	/
Coxsackie B virus antibody IgG	Positive	Negative	/
EB virus VCA antibody IgG	Positive	Negative	/
α1-microglobulin, mg/L	21.14	41.90	5.77
β2-microglobulin, μg/L	1010.0	4366.4	100.0
Microalbumin, mg/L	1259.3	1327.6	740.2
Urinary transferrin, mg/L	97.90	123.95	43.39
Urinary IgG, mg/L	13.52	24.63	81.5
N-acetyl-β-D-glucosaminidase, U/L	34.7	28.1	6.5

IgG: immunoglobulin G.
